# Renal tubular acidosis, food allergy, IgA and IgG deficiency in Mexican children

**DOI:** 10.1186/2045-7022-1-S1-P111

**Published:** 2011-08-12

**Authors:** Elizabeth Estrada-Reyes, Blanca Morfin Maciel, Mara Medeiros-Domingo

**Affiliations:** 1 Hospital Angeles Metropolitano, Allergy, Mexico City, Mexico; 2 Hospital Angeles Mocel, Allergy, Mexico City, Mexico; 3 Hospital Angeles Metropolitano, Nephrology, Mexico City, Mexico

## Background

Although. recurrent upper airways infections along with gastrointestinal symptoms seen in patients with Selective IgA deficiency and IgG4. In Mexican children population we have found a link between food allergy and renal tubular acidosis, being a food allergen as the trigger for both diseases. And an immunodeficiency patient is a high risk to develop.

## Aim

To describe the association between food allergy, renal tubular acidosis and IgA and IgG deficiency, there is a serial case description.

## Material and Methods

We report 30 patients attended at our hospital between January 2008 and September 2010 who were diagnosed with food allergies, renal tubular acidosis and IgA /IgG4 deficiency. Food allergy was confirmed by an oral challenge to cow's milk, soy, egg, wheat and corn in addition to IgE levels for cow's milk, egg, soy, wheat and corn as well as patch test. IgA deficiency was considering as serum IgA levels below 5 mg per dl, IgG4 levels below the percentile described according to the patient age. Complete blood cell count (CBC) were performed in all patients being in normal levels. Renal tubular acidosis considering a HCO3 level below 21 electrolyte and urinary criteria.

## Results

Median age 20 months (±12 months), 15 female (50%) 15 male(50%). IgA2 deficiency 16 (53%), IgG4 and IgA2 deficiencies 13 (43%), IgG4 deficiency 27 (90%), 29 patients had distal renal tubular acidosis (96%) 1 patient with proximal renal tubular acidosis. About food allergies, cow's milk 28(93%), egg 18 (60%), wheat 0, corn 8 (26.6%), soy 2 (6.6%), non IgE mediated to cow's milk 6 (20%), egg 6 (20%), soy 7 (23%), wheat 4 (13%), corn 1 (3.3%). The formula used to cow's milk allergy was aminoacid based formula 19 (63.3%), extensively hydrolized formula 11 (36.6%). Gastroesophageal reflux was associated in 83.3%, wheezing 75%, diarrhea 33.3%, atopic dermatitis 25%, rhinitis 83.3%, urticaria 19.4%, constipaciòn 2.8%, blood stools 2.8%.

## Discussion

There is quite a few reports about food allergy and renal tubular acidosis, as an allergen trigger of the mechanism of acidosis production.

## Conclusion

Is very important to observe not just the cow's milk.

